# Dual-color image analysis for quantifying fluorescence intensity in plasma membrane region of cells

**DOI:** 10.1007/s44211-026-00908-y

**Published:** 2026-04-14

**Authors:** Satoshi Fujii, Keita Takaki, Shinji Sueda

**Affiliations:** Department of Bioscience and Bioinformatics, Kyushu Institute of Technology, 680-4, Kawazu, 820-8502 Iizuka, Japan

**Keywords:** Plasma membrane fluorescence, Image analysis, Cell segmentation, Ratiometric quantification, Web application

## Abstract

Quantitative evaluation of fluorescence intensity in the plasma membrane region is essential for a wide range of cell-based imaging studies, including membrane protein analysis and cell surface interaction assays. However, accurate quantification remains challenging because membrane regions are often manually selected, leading to subjective variabilities and limited reproducibility. Here, we developed a dual-channel image analysis framework and its web-based tool, DualCellQuant, which enables the objective and reproducible quantification of fluorescence intensity in the plasma membrane region. This method integrates automated cell segmentation using Cellpose-SAM with Euclidean distance transform-based radial normalization to define membrane regions at a fixed normalized distance from the cell boundary. The membrane region was determined on a reference channel, allowing robust quantification even for low-intensity target signals. In addition, ratiometric analysis between channels can mitigate cell-to-cell variability. We applied this approach to quantify rapamycin-induced binding between FKBP12 displayed on the cell surface and fluorescein-labeled FRB, demonstrating that membrane-associated fluorescence signals can be consistently extracted on a per-cell basis and used for binding-equilibrium analysis. This accessible framework is expected to facilitate the reproducible interpretation of multicolor cellular imaging data across diverse experimental settings.

**Graphical abstract:**

**Supplementary Information:**

The online version contains supplementary material available at 10.1007/s44211-026-00908-y.

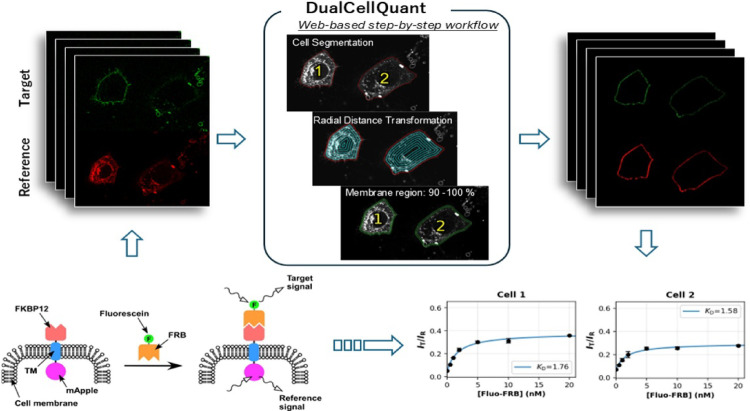

## Introduction

Fluorescence imaging of cells is an indispensable technique for elucidating a wide range of biological phenomena and cell-based bioanalytical applications [1]. Among these, imaging of the plasma membrane region has been widely utilized for tracking cell morphology [2], functional analysis of membrane proteins [3], drug discovery [4], and biosensing at the cell surface [5]. A critical requirement for plasma membrane imaging is the quantitative evaluation of fluorescence intensity within the membrane region. However, the quantitative analysis of membrane-associated fluorescence generally requires manual or semi-manual selection of membrane regions [6], and the resulting intensity values are often highly sensitive to the choice of regions. This subjectivity makes it difficult to obtain reproducible and objective quantitative data from the images. Several computational tools have been proposed to facilitate plasma membrane intensity quantification; nevertheless, robust and consistent analysis remains challenging across diverse imaging conditions [6]. In this study, we developed an image analysis method that enables the objective quantification of fluorescence intensity in the plasma membrane region, based on Cellpose, a recently developed automated cellular segmentation program, and its latest extension, Cellpose-SAM [7, 8].

**Fig. 1:**
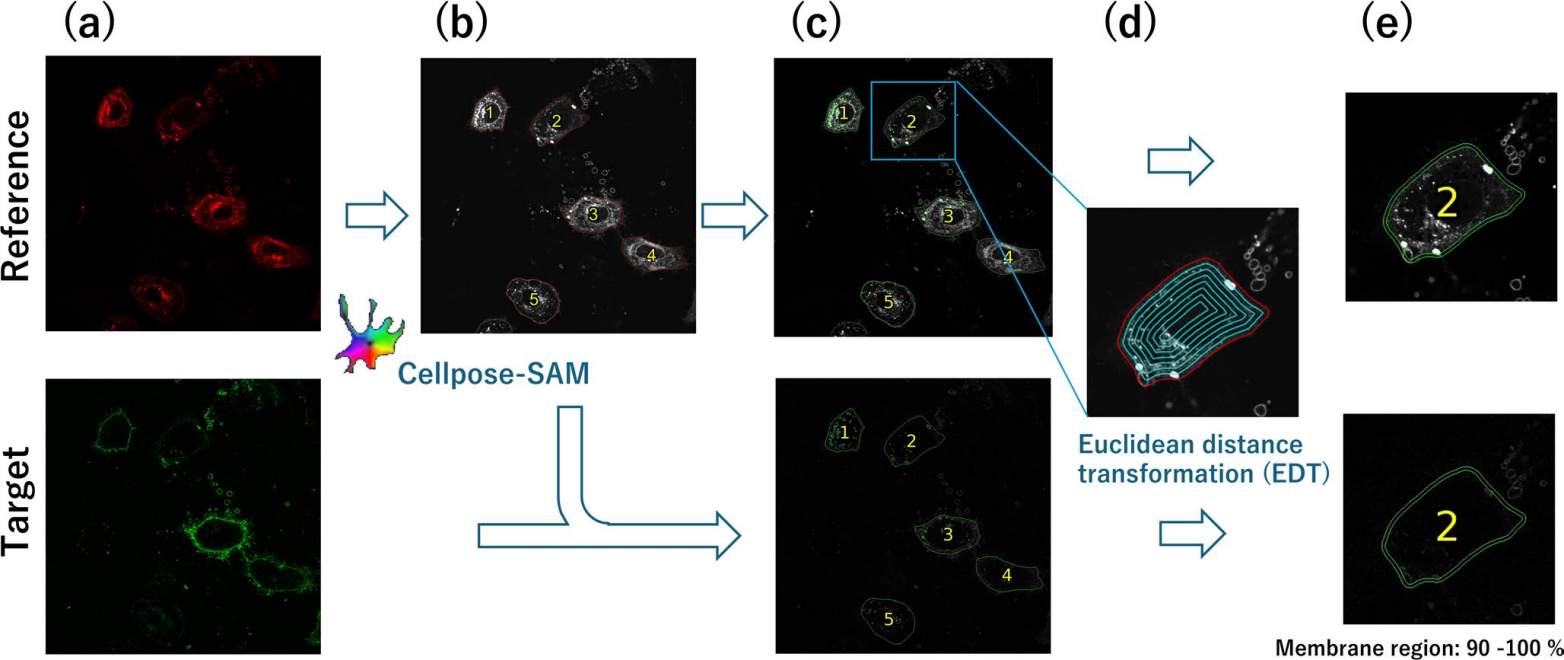
Overview of the image analysis workflow for quantifying the fluorescence intensity in the plasma membrane region. **a** Raw image data from the reference channel (top) and the target channel (bottom). **b** Cell segmentation and numbering using Cellpose-SAM. **c** Mask creation for the target and reference channels, and extraction of common regions. **d** Radial distance transformation based on the Euclidean distance transform (EDT). The red contour line indicates the cell boundary, and the cyan contour lines indicate the equal normalized distances from the cell boundary. **e** Definition of the plasma membrane region and fluorescence intensity measurements. The membrane region was defined as *r* = 90–100% (green band)

To evaluate the fluorescence intensity in the plasma membrane region, individual cells must first be identified and segmented. Automated cell segmentation has become indispensable for quantitative microscopy analysis, enabling the accurate delineation of individual cells for downstream phenotypic and morphological quantification. Deep learning-based approaches have substantially improved segmentation performance compared with classical thresholding and watershed methods [9, 10]. However, many deep learning-based segmentation methods still require training that is tailored to specific cell types or experimental conditions. Recently, Cellpose-SAM has been developed by integrating the Segment Anything Model (SAM) with Cellpose [7, 8, 11], enabling robust cell segmentation across a wide variety of cell types without the need for additional training. In this study, we employed Cellpose-SAM to identify cell contours and propose an image analysis method (its web-based application; DualCellQuant) that quantitatively evaluates the fluorescence intensity in the plasma membrane region based on the extracted boundary information (Fig. 1).

In our method, the plasma membrane region is defined based on the normalized distance from the cell boundary using a Euclidean distance transform (EDT) [12], and the fluorescence intensity within this region is quantitatively evaluated. By selecting the membrane region at a fixed proportion relative to the cell contour, the analysis can be performed objectively and consistently across different cells. In addition, the proposed system was designed to handle multicolor imaging data by simultaneously processing two fluorescence channels. Even when the fluorescence intensity in one channel is weak, the membrane region can be reliably determined based on the channel with stronger fluorescence, allowing the quantitative evaluation of membrane-associated signals in the low-intensity channel. Furthermore, one fluorescence channel can be used as a reference to normalize or correct the intensity of another channel.

**Fig. 2:**
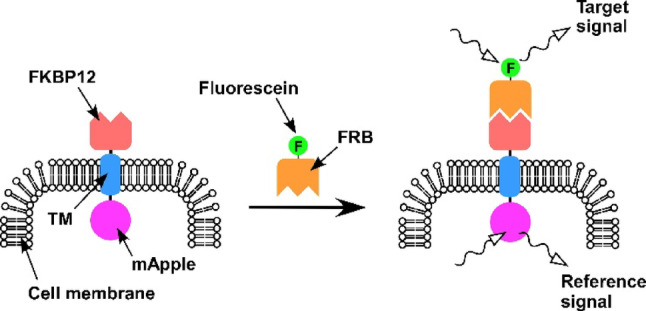
Schematic illustration of protein-protein interaction analysis on the cell surface. The binding between FKBP12 and FRB was used as a model for protein-protein interactions. FKBP12 was displayed on the cell surface by expressing it as a fusion protein with TM carrying mApple on the cytoplasmic side of the membrane. Fluorescein-labeled FRB was added to the cells, and its binding to FKBP12 was observed under a fluorescence microscope to analyze the binding between the two proteins. In the fluorescence observation, the fluorescent signal derived from fluorescein (target signal) was observed together with the fluorescent signal derived from mApple (reference signal). Although rapamycin is required for the binding between FKBP12 and FRB, it is omitted from this figure for simplicity

To demonstrate the utility of our image analysis method, we applied it to the analysis of protein–protein interactions on the cell surface using fluorescence imaging (Fig. 2). In this system, one of the interacting proteins is expressed on the cell surface as a fusion protein with a membrane protein, whereas the other binding partner is fluorescently labeled and added to the extracellular medium. Fluorescence imaging data were acquired using confocal laser scanning microscopy (CLSM), and the binding level of the partner protein on the cell surface was quantitatively evaluated. As a proof-of-concept study to validate the analytical capability and flexibility of this tool, we employed the interaction between FKBP12 and FRB, whose association is induced by the antibiotic rapamycin [13, 14]. FKBP12 is expressed on the cell surface as a fusion protein with the transmembrane domain (TM) of the platelet-derived growth factor receptor. To visualize the expression of the fusion protein, a red fluorescent protein, mApple, was fused to the cytoplasmic side of the TM domain; this fusion protein was designated FKBP12–TM–mApple. For the binding partner, a recombinant FRB protein containing a cysteine ​​residue introduced near the N-terminus was prepared and labeled with fluorescein, and the labeled protein was designated as Fluo-FRB.

In the binding experiments, a series of Fluo-FRB concentrations was sequentially added to cells expressing FKBP12–TM–mApple, and fluorescence images were acquired at each concentration. The fluorescence intensity in the plasma membrane region was estimated using our image analysis method, and the binding level of Fluo-FRB was evaluated. Binding curves were generated from these data, and their accuracy was assessed to demonstrate the applicability of our method. In addition, the effectiveness of normalization based on ratiometric analysis with two-channel fluorescence images was examined.

## Experimental

### Materials

The plasmid, pmCheery-FKBP12, carrying the gene of FKBP12, and the plasmid, pEGFP-FRB, carrying the gene of FRB, were gifts from Dorus Gadella (Addgene plasmid # 67900) [15] and from Klaus Hahn (Addgene plasmid # 25919) [16], respectively. Oligonucleotides used as PCR primers were custom-synthesized by Gene Design (Osaka, Japan). HeLa cells were obtained from the JCRB Cell Bank (Ibaraki, Japan). The X-tremeGENE 9 DNA transfection reagent was purchased from Roche (Basel, Switzerland). The glass-bottom dishes pre-coated with collagen were purchased from Matsunami Glass (Osaka, Japan). Fluorescein-5-maleimide was sourced from Tokyo Chemical Industry (Tokyo, Japan). Other common chemicals and materials were obtained from local suppliers.

## Construction of the expression plasmid for FKBP12-TM-mApple

The expression plasmid for FKBP12-TM-mApple in mammalian cells was constructed using pBCCP-TM-mApple prepared in the previous study [17] and pmCheery-FKBP12 obtained from Addgene. Detailed procedures are described in the Supplementary Information.

## Preparation of Fluo-FRB

A recombinant FRB protein with a cysteine ​​residue introduced near the N-terminus (Cys-FRB) was prepared by constructing its expression system in *Escherichia coli* and purifying it from *E. coli* cells. The expression plasmid was prepared by modifying the FRB gene derived from pEGFP-FRB (from Addgene) and inserting it into the pET21a vector. Detailed procedures are described in the Supplementary Information. Cys-FRB was purified from *E. coli* BL21(DE3) transfected with its expression plasmid by nickel affinity chromatography according to standard procedures. Fluo-FRB was then prepared by modifying the cysteine ​​residues of the purified Cys-FRB with fluorescein-5-maleimide.

## Cell culture and fluorescence imaging of cells

In this work, HeLa cells were used for all experiments; they were cultured in Dulbecco’s Modified Eagle’s Medium (DMEM) supplemented with 10% fetal bovine serum at 37 °C under 5% CO_2_. FKBP12-TM-mApple was expressed in HeLa cells by introducing its expression plasmid into the cells by DNA transfection. Specifically, the cells were seeded on a 35-mm glass-bottom dish coated with collagen and cultured for approximately 24 h. The cells were then transfected with the expression plasmid for FKBP12-TM-mApple using X-tremeGENE 9 DNA transfection reagent according to the manufacturer’s protocol. Twenty-four hours after transfection, the cells were washed with PBS, and DMEM containing Fluo-FRB and rapamycin was added to the cells under the respective experimental conditions. The cells were then observed by confocal laser scanning microscopy (CLSM) on a Fluoview FV1200 system (Olympus, Tokyo, Japan). Fluorescence from fluorescein was monitored using the green channel with excitation at 473 nm and detection from 490 to 540 nm, and fluorescence from mApple was monitored using the red channel with excitation at 559 nm and detection from 575 to 675 nm. Each image was taken with a resolution of 0.207 μm per pixel.

## Binding equilibrium experiments

In the binding equilibrium experiments, Fluo-FRB was added successively at different concentrations (final concentrations of 0.5–20 nM) to cells expressing FKBP12-TM-mApple in the presence of 100 nM rapamycin, and images were taken under each concentration condition. Here, images were first taken before adding Fluo-FRB, and then images were taken after adding Fluo-FRB at each concentration and incubating for 10 min. In this in situ binding equilibrium assay, washing steps were omitted, which meant that unbound Fluo-FRB remained in the bulk extracellular solution. Therefore, CLSM was essential for optically sectioning the cells and exclusively monitoring the fluorescence at the focal plane, eliminating the strong background from the out-of-focus bulk solution. When acquiring images, four-slice images were taken in the focal planes at different depths with an interval of 0.5 μm under each concentration condition. The data obtained under each Fluo-FRB concentration condition were subjected to image analysis to evaluate the binding level of Fluo-FRB. Specifically, the fluorescence intensity values on the membrane region were quantified as described below.

### Quantification of fluorescence intensity in the plasma membrane region

An overview of the analysis workflow for quantifying the fluorescence intensity in the plasma membrane region is shown in Fig. 1. In this method, two-channel fluorescence microscopy images were used as inputs, where the fluorescence image derived from fluorescein was treated as the target channel, and that derived from mApple was treated as the reference channel. Based on the automatic cell segmentation of the reference images and Euclidean distance transform-based analysis, the fluorescence intensity values in the plasma membrane region were quantitatively evaluated on a per-cell basis. The present analysis method is available as a web application, DualCellQuant (https://dna00.bio.kyutech.ac.jp/dualcellquant/), which allows users to reproduce the analysis procedure step-by-step while interactively adjusting various parameters through a web browser. In addition, the source code is publicly available on GitHub (https://github.com/fuji3to4/DualCellQuant). The core functions can be imported as a Python library and integrated into user-defined workflows. The details of each analysis step are provided below.

1. Cell segmentation

Individual cells were segmented in the reference channel images using Cellpose-SAM software (Fig. 1a, b). The segmentation parameters for Cellpose-SAM were set to a diameter of 100, flow threshold of 0.4, and cell probability threshold of 1.0 [8]. These parameters were empirically determined by comparing multiple conditions to minimize over- and under-segmentation across all microscopy images used in this study. Each segmented cell was assigned a unique identifier (ID). No independent segmentation was performed for the target channel; instead, the segmentation masks obtained from the reference channel were directly applied. Cells contacting the image boundaries were excluded from further analysis based on the geometric criteria. Specifically, a margin region corresponding to 1% of the image dimensions was defined along the image borders, and any segmented cell for which more than 2% of its pixels fell within this margin region was excluded from analysis.

(2) Target/reference mask creation

Masks were generated for both the target and reference channels to extract the pixel intensity values from each measurement channel (Fig. 1c). Saturated pixels (8-bit intensity value of 255) were excluded from the analysis, and mask regions smaller than 50 pixels were removed as noise. The masks from the target and reference channels were then combined by taking their intersection, and only the pixel intensity values within the regions present in both channels were included in the subsequent analysis.

(3) Radial distance transformation

To define the plasma membrane region for each cell using an objective and consistent criterion, a normalized distance metric based on the distance from the cell boundary was established. Specifically, a Euclidean distance transform was applied to quantify the spatial positions inside and outside the cell relative to the cell boundary [12, 18] (Fig. 1d). For each cell, the segmented cell region mask was used as a foreground mask, and a Euclidean distance transformation was applied. Consequently, the Euclidean distance *d* from the cell boundary was calculated for each pixel inside the cell. This distance *d* reaches its maximum near the cell center and decreases toward the cell boundary (Fig. S1). The maximum distance within each cell was defined as *d*_*max*_, and the normalized radial distance *r* was defined as the following Eq. (1):

1 $$r=100\times\left(1-\frac{d}{{d}_{max}}\right)$$

With this definition, *r* = 0% corresponds to the location of the maximum distance transform (approximately the cell center), whereas *r* = 100% corresponds to the cell boundaries. For the extracellular region, the Euclidean distance transform was similarly applied to the background mask, and the distances from the cell boundary were normalized using the same scale, thereby defining regions with *r* > 100%. In this study, regions up to *r* = 150% were included in the analyses. Based on the normalized radial distance *r*, the plasma membrane region was defined using the inner (*r*_inner_) and outer (*r*_outer_) boundary parameters. In this study, *r*_inner_ was set to 90% and *r*_outer_ to 100%, and pixels satisfying 90% ≤ *r* ≤ 100% were defined as the membrane-region mask for each cell (Fig. 1e).

(4) Intensity measurement

For both the target and reference channels, the mean intensity of the pixels within the membrane region mask was calculated and defined as *I*_T_ (target) and *I*_R_(reference), respectively. Background correction was performed by calculating the mean intensity of pixels with intensity values in the lowest 5% of each image, which was defined as the background intensity and was subtracted from all pixel intensities. In addition, the target-to-reference intensity ratio *I*_T_/*I*_R_ was calculated.

(5) Radial profile calculation

The fluorescence intensity distribution from the cell center to the extracellular region was calculated using the sliding window approach. Within the normalized distance range of 0–150%, annular regions parallel to the cell boundary were defined and sequentially shifted inward and outward for the analysis. Specifically, annular regions with a width corresponding to 5% of the normalized distance (e.g., 95–100% and 93–98%) were defined and shifted in steps of 2%. The mean fluorescence intensity of the pixels included in each annular region was calculated. Thus, a one-dimensional radial profile representing the fluorescence intensity distribution from the cell center to the extracellular region was obtained for each cell. To reduce noise, the resulting radial profiles were smoothed using a Savitzky–Golay filter with a window size of 5 and a polynomial order of 2 [19].

### Binding curve fitting and *K*_D_ estimation

Binding curves were generated by plotting the binding levels against Fluo-FRB concentrations. As binding level criteria, two types of values were used: the mean membrane intensity derived from the fluorescein channel (*I*_T_) and the intensity ratio between the fluorescein and mApple channels (*I*_T_/*I*_R_). Data based on each metric were independently subjected to binding analysis. Although the formation of the FKBP12-rapamycin-FRB complex involves three components, previous studies have demonstrated that the dissociation constant (*K*_D_) between FKBP12 and rapamycin is 0.2 nM, whereas the *K*_D_ between FRB and rapamycin is 26 µM [13]. Therefore, under our experimental conditions using 100 nM rapamycin, FKBP12 forms a complex with rapamycin almost entirely, whereas FRB essentially exists in a free state in the bulk solution. Consequently, the binding process can be regarded as a bimolecular interaction between the FKBP12–rapamycin complex and FRB. The dissociation constant (*K*_D_) values ​​were calculated by nonlinear regression analysis of the binding curves using the following Langmuir Eq. (2):

2 $$BL=\frac{{BL}_{\mathrm{m}\mathrm{a}\mathrm{x}}\left[FRB\right]}{{K}_{\mathrm{D}}+\left[FRB\right]}+BG$$

In Eq. (2), *BL* represents the binding level of Fluo-FRB at a given concentration, and BG denotes the background level corresponding to the intensity level in the absence of Fluo-FRB. [*FRB*] represents the concentration of Fluo-FRB. *K*_D_ and *BL*_max_ represent the dissociation constants of the FRB-FKBP12 complex and the maximum binding level of Fluo-FRB, respectively, as estimated by regression analysis. Using both *I*_T_ and *I*_T_/*I*_R_ values ​​as binding levels, fitting analysis was performed based on Eq. (2).

## Results and discussion

### Expression of FKBP12-TM-mApple and its binding with Fluo-FRB

**Fig. 3:**
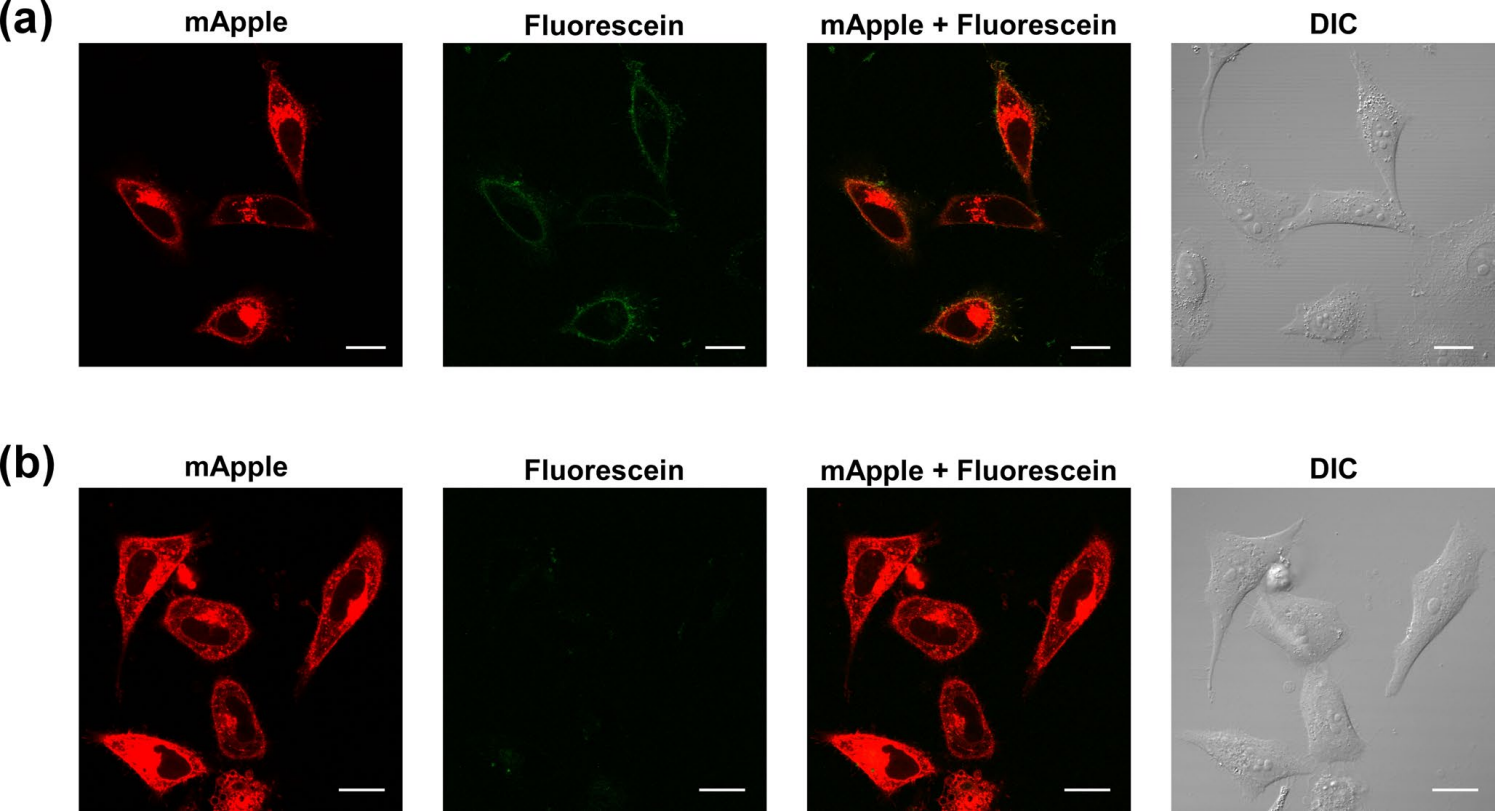
Expression of FKBP12-TM-mApple and its binding with Fluo-FRB. HeLa cells were transfected with an expression plasmid for FKBP12-TM-mApple, and 24 h after transfection, the cells were observed in the presence of 50 nM Fluo-FRB and 100 nM rapamycin by CLSM (**a**). A similar experiment was performed in the absence of rapamycin (**b**). The leftmost panels show fluorescence images derived from mApple, and the second panels from the left show fluorescence images derived from fluorescein. The second panels from the right show overlays of both fluorescence images, and the rightmost panels show differential interference contrast (DIC) images. Scale bars represent 20 μm

We first confirmed the expression of FKBP12-TM-mApple and its binding with Fluo-FRB on the cell surface. Thus, HeLa cells were transfected with the expression plasmid of FKBP12-TM-mApple, and then, 24 h after transfection, the cells were observed in the presence of Fluo-FRB and rapamycin by CLSM. As shown in Fig. 3a, red fluorescence derived from mApple was observed from the periphery of the cells, confirming that FKBP12-TM-mApple was expressed in the cells and most of it was translocated to the plasma membrane. Furthermore, green fluorescence from fluorescein was observed at the periphery of the cells, where mApple-derived fluorescence was observed, confirming that Fluo-FRB was bound to the cell surface. In contrast, when a similar experiment was performed without rapamycin, fluorescence from fluorescein was scarcely observed around the cells (Fig. 3b). This indicates that the fluorescence from fluorescein observed around the cells in the presence of rapamycin is due to the specific binding between FKPB12 and FRB, demonstrating that this experimental system can be used to confirm the binding between these two proteins.

### Experimental data for binding equilibrium experiments

**Fig. 4:**
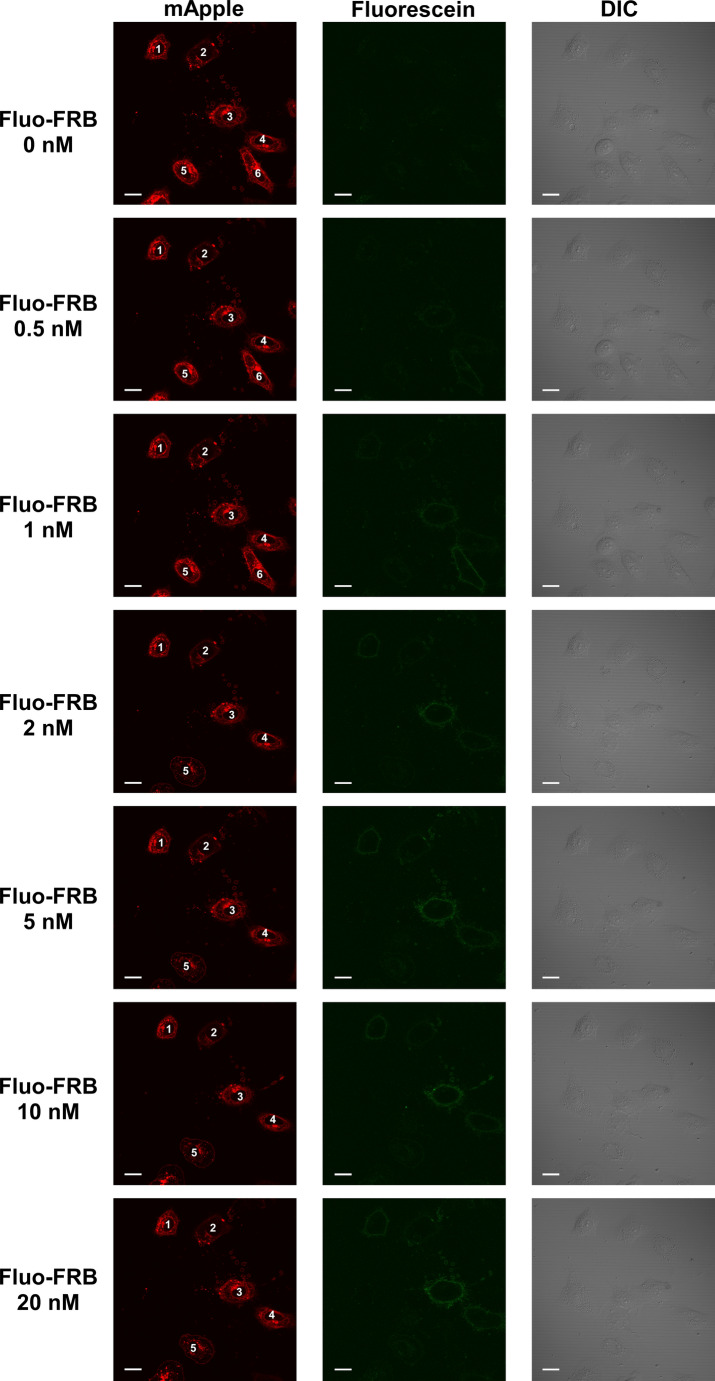
A series of images of cells expressing FKBP12-TM-mApple after the addition of different concentrations of Fluo-FRB. Fluo-FRB was added sequentially to HeLa cells expressing FKBP12-TM-mApple in the presence of rapamycin at different concentrations ranging from 0.5 nM to 20 nM, and fluorescence images were taken at each concentration of Fluo-FRB. Four-slice images were acquired under each Fluo-FRB concentration condition, and one series of image data at the same focal plane is shown here. The left column shows mApple-derived fluorescence images, the center column shows fluorescein-derived fluorescence images, and the right column shows differential interference contrast (DIC) images. The IDs of the cells assigned in the image analysis are shown in the images derived from mApple. The scale bars represent 20 μm

Next, to perform the binding equilibrium analysis between FKBP12 and FRB, we successively added a series of Fluo-FRB concentrations to cells expressing FKBP12-TM-mApple in the presence of rapamycin and acquired fluorescence image data under each concentration condition. Four-slice images were acquired in the focal planes at different depths for each concentration. Figure 4 shows a set of image data obtained at a series of Fluo-FRB concentrations at the same focal planes. The original data for all images, including those at the other focal planes, is publicly available (see Data availability). This shows that the intensity of fluorescence derived from fluorescein observed around the cells increased with increasing Fluo-FRB concentration. As clearly seen from the fluorescence image data derived from mApple in each cell, the fluorescence intensity level depends on the expression level of the fusion protein and cell shape. Therefore, we separately evaluated the binding levels of Fluo-FRB in each cell and performed a binding analysis for each cell. The IDs of the cells assigned in the image analysis are shown in the images derived from mApple (Fig. 4). Although the concentration-dependent increase in fluorescence from fluorescein at the plasma membrane may appear subtle to the naked eye in microscopic images, our image analysis method accurately extracts these faint signals for robust quantification, as described below.

### Determination of the plasma membrane region based on radial profiles

**Fig. 5:**
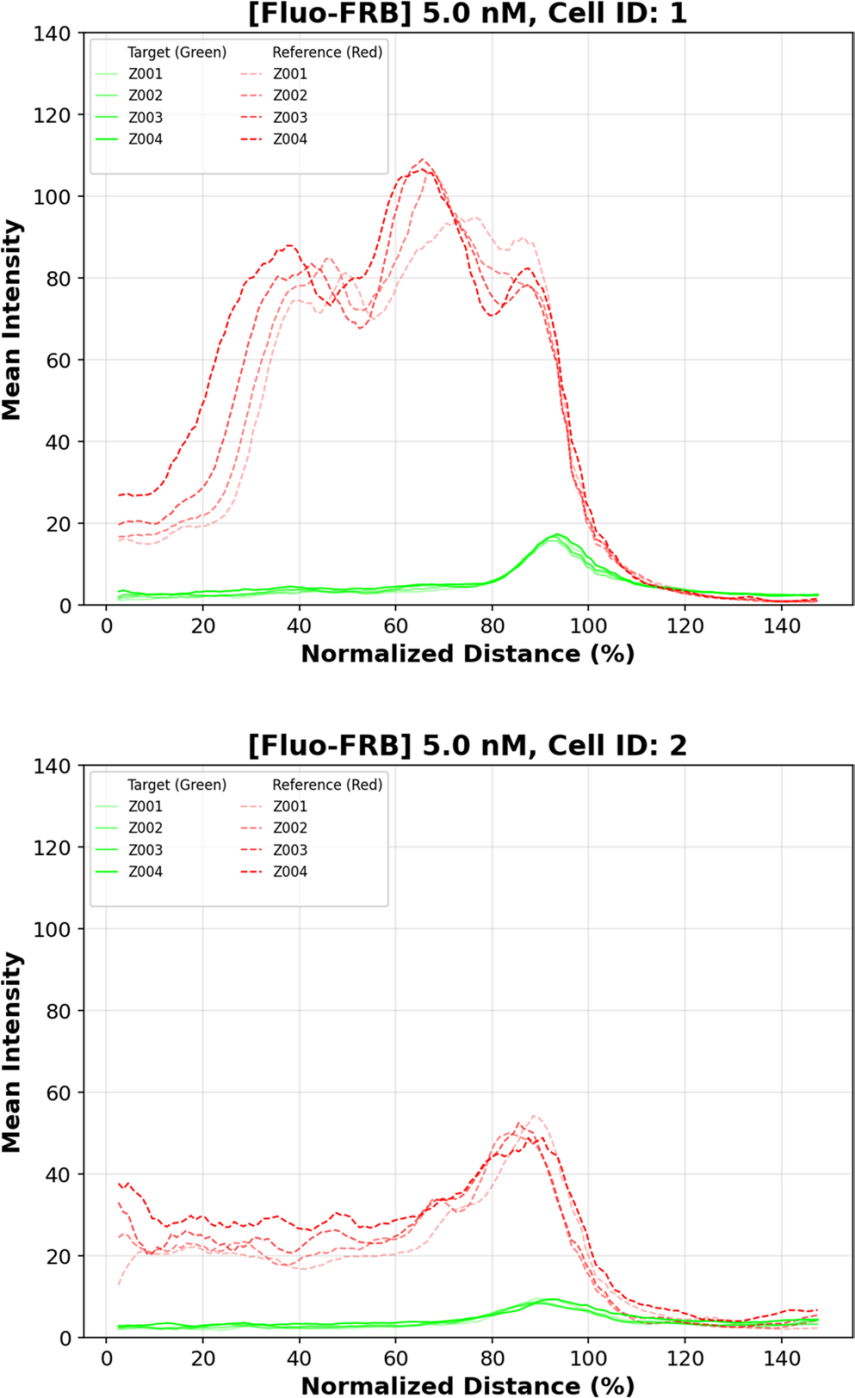
Representative radial fluorescence intensity profiles of the cells. Radial profiles of the mean fluorescence intensity are shown as a function of the normalized distance from the cell center to the extracellular region for Cell IDs 1 (top) and 2 (bottom) at a Fluo-FRB concentration of 5.0 nM. The green solid lines represent the profiles from the target channel (Fluo-FRB), and the red dashed lines represent those from the reference channel (mApple). Profiles obtained from the four z-slice images (Z001–Z004) are shown for each cell

The range of the plasma membrane region used for subsequent quantitative analysis was determined by referring to fluorescence intensity profiles based on normalized radial distance (radial profiles). Radial profiles represent the spatial distribution of fluorescence intensity from the cell center to the cell boundary and the extracellular region. Figure 5 shows the representative radial profiles for Cell IDs 1 and 2 obtained under conditions where Fluo-FRB was added at a concentration of 5.0 nM. The radial profiles of all analyzed cells are shown in Fig. S2.

In the reference channel derived from mApple fluorescence, a clear intensity peak was observed near a normalized distance of 90%, corresponding to the cell boundary. The fluorescence signal exhibited a relatively broad distribution that extended toward the cell interior. This distribution likely reflects the fluorescence signals from FKBP12-TM-mApple distributed at the outer nuclear membrane and endoplasmic reticulum membrane before transport to the plasma membrane. In contrast, the fluorescence from the target channel (Fluo-FRB) exhibited a localized peak near a normalized distance of 90%, with a negligible detectable signal inside the cell. This distribution is consistent with the extracellular addition of Fluo-FRB and its binding to FKBP12 on the cell surface.

The shapes and intensities of the radial profiles varied among individual cells. In the reference channel, substantial cell-to-cell variability was observed, particularly in the extent of signal spreading toward the cell interior and the peak sharpness. This variability is likely attributable to differences in the transient expression levels and intracellular transport states of the FKBP12-TM-mApple fusion protein among individual cells, resulting in varying amounts of fluorescent protein being retained in intracellular secretory compartments. In contrast, the fluorescence in the target channel consistently exhibited a single membrane-associated peak in most cells.

In this analysis, the normalized radial distance of 100% was defined as the cell boundary determined by automated cell segmentation. In the radial profiles, however, the fluorescence intensity maxima in both channels were not observed exactly at 100% but slightly inside the cell, with peaks located near a normalized distance of 90%. This shift likely reflects the combined effects of membrane thickness, optical point spread function, and averaging of pixel intensities within the segmented boundary. Based on these observations, the normalized radial distance range of 90–100% was defined as the plasma membrane region and used for subsequent quantitative analyses.

### Binding analysis based on fluorescence intensity in the plasma membrane region

For each Fluo-FRB concentration condition, image analysis was performed to quantify *I*_T_ (target channel), *I*_R_ (reference channel), and their ratio (*I*_T_/*I*_R_). Binding curves were generated by plotting *I*_T_ or *I*_T_/*I*_R_ against the Fluo-FRB concentration, and nonlinear regression analysis based on the Langmuir isotherm was conducted to estimate the binding parameters. In this analysis, cells that were partially located outside the image boundaries under any Fluo-FRB concentration condition were excluded. Consequently, only those cells that could be consistently tracked across all Fluo-FRB concentrations were included. Specifically, four cells (Cell IDs 1, 2, 3, and 5) were analyzed across seven different concentration points using four z-slice images at each point, resulting in a total of 112 images. The membrane intensity values were obtained by averaging the four z-slice images for each concentration within each individual cell.

**Fig. 6:**
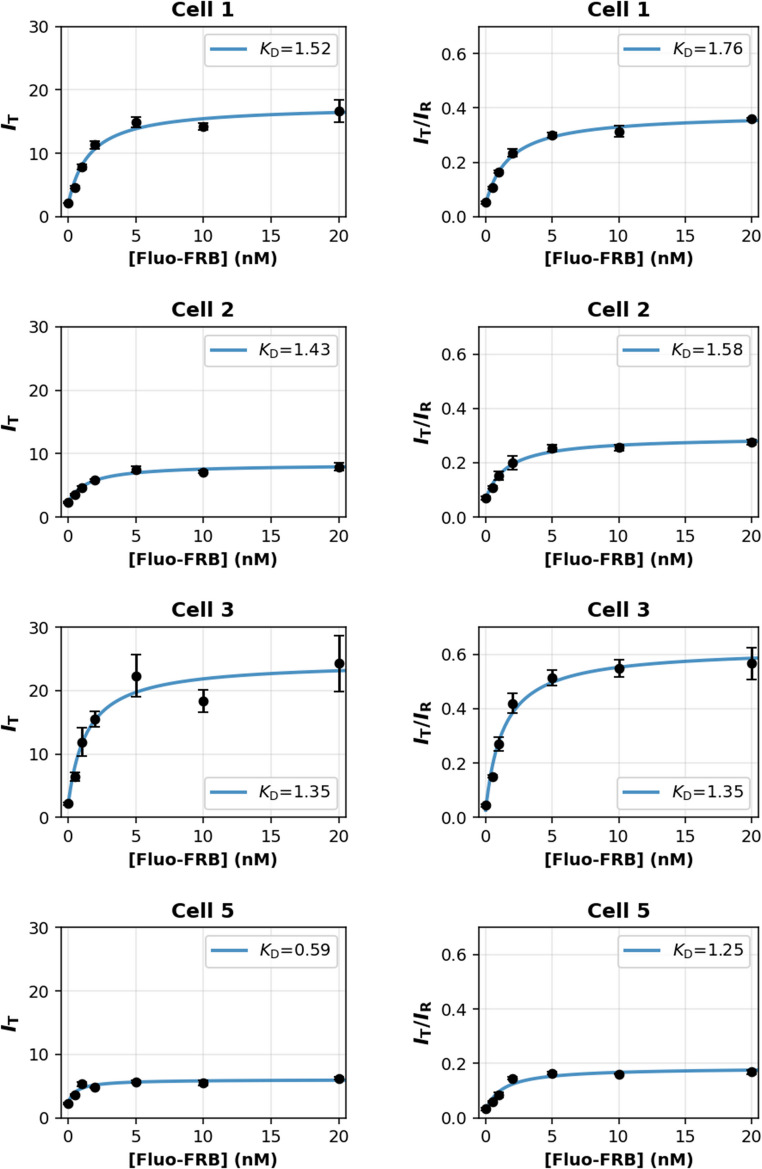
Binding curves between FKBP12 and FRB on the cell surface. Binding curves were generated on individual cells (Cell IDs 1, 2, 3, and 5) by plotting the mean fluorescence intensity from the target channel (*I*_T_) or the ratio (*I*_T_/*I*_R_) of *I*_T_ to that from the reference channel (*I*_R_) against the Fluo-FRB concentration; the former curves are shown in the left column and the latter curves are shown in the right column. Each curve was fitted to the Langmuir Eq. (2). Data points represent mean values obtained from four z-slice images for each Fluo-FRB concentration, and the error bars indicate standard deviations. Solid lines indicate nonlinear regression fits, and the estimated dissociation constants (*K*_D_) in nanomolar concentration are shown in each panel

Saturable binding curves consistent with the Langmuir isotherm were obtained using either *I*_T_ or *I*_T_/*I*_R_ as the binding metric (Fig. 6). The estimated dissociation constants (*K*_D_) and fitting statistics are summarized in Table 1. High coefficients of determination (*R*^2^) were obtained for both metrics (*I*_T_: 0.92–0.98; *I*_T_/*I*_R_: 0.95–0.99), indicating that the membrane fluorescence intensities derived from image analysis were suitable for quantitative binding equilibrium analysis. The *K*_D_ values (means ± standard deviation, *n* = 4 cells) were 1.22 ± 0.43 nM for *I*_T_-based analysis and 1.48 ± 0.23 nM for *I*_T_/*I*_R_-based analysis, respectively. Notably, the use of *I*_T_/*I*_R_ tended to yield more consistent *K*_D_ values with less variability.

**Table 1:** Dissociation constants (*K*_D_) and fitting statistics obtained from the Langmuir binding analysis^a^

Cell ID		*I*_T_			*I*_T_/*I*_*R*_	
	*K*_D_ (nM)^b^	SE^b^ of *K*_D_	*R* ^2b^	*K*_D_ (nM)^b^	SE^b^ of *K*_D_	*R* ^2b^
1	1.52	0.45	0.976	1.76	0.34	0.990
2	1.43	0.40	0.978	1.58	0.32	0.989
3	1.35	0.61	0.943	1.35	0.31	0.985
5	0.59	0.31	0.918	1.25	0.55	0.946
Means ± SD^c^	1.22 ± 0.43			1.48 ± 0.23		

^a^Langmuir isotherm fitting was performed on a per-cell basis using either the mean fluorescence intensity from the target channel (*I*_T_) or the ratio (*I*_T_/*I*_R_) of *I*_T_ to that from the reference channel (*I*_R_) as the binding metric

^b^*K*_D_ values, standard errors of *K*_D_, and coefficients of determination (*R*^2^) were estimated using nonlinear regression analysis of the binding curves shown in Fig. 6

^c^The means ± standard deviations from data on four cells are shown

These results demonstrate that binding equilibrium analysis can be conducted using quantitative data obtained by our image analysis method, enabling the evaluation of binding properties at the level of individual cells. For comparison, previous biochemical studies using purified proteins have reported *K*_D_ values for the interaction between the FKBP12-rapamycin complex and FRB as 12 nM via surface plasmon resonance (SPR) [13] and approximately 5 nM via an affinity-binding assay [20]. The apparent *K*_D_ values obtained in our study (1.2–1.8 nM) are in reasonable agreement with these values, considering the differences in the assay environments (e.g., in situ batch assay on the living cell membrane versus in vitro flow or column assays).

### Significance of the membrane-specific region extraction

To validate the necessity of specifically extracting the plasma membrane region, we performed a comparative analysis using the fluorescence intensity of the entire cell region (i.e., using the full Cellpose-SAM masks without EDT-based boundary restriction). As shown in Fig. S3 and Table S1, quantifying the entire cell region resulted in substantially larger variability in the calculated *K*_D_​ values and lower fitting accuracy (*R*^2^) compared to the membrane-restricted analysis. This is likely because whole-cell analysis incorporates variable intracellular background noise. These results demonstrate that strictly isolating the membrane region is highly appropriate and necessary for the reliable quantification of membrane-associated interactions.

### Significance of the ratiometric analysis

In the binding analyses described above, ratiometric analysis tended to reduce focal-plane dependence and cell-to-cell variability, leading to a more stable binding evaluation. At the same time, the effectiveness of ratiometric analysis depends on the measurement conditions and sample characteristics. As shown in Fig. 6, for Cell ID 3, large error bars were observed when *I*_T_ alone was used, whereas the variability was reduced when the ratio (*I*_T_/*I*_R_) was used. Consistently, the coefficient of determination (*R*^2^) for this cell increased from 0.94 with *I*_T_ alone to 0.98 with *I*_T_/*I*_R_. Similarly, for Cell ID 5, the relatively low expression level of the reference protein resulted in a low overall target intensity, leading to an underestimated *K*_D_ value of 0.59 nM when *I*_T_ was used alone. However, normalization via *I*_T_/*I*_R_ effectively corrected this expression-level variation, yielding a *K*_D_ ​value of 1.25 nM, which was reasonably consistent with those of the other cells. These results indicate that for cells strongly affected by membrane boundary detection errors or focal plane dependence, normalization on the reference channel can reduce variability and improve the robustness of binding curve fitting. In contrast, if the reference channel exhibits large intensity fluctuations, ratiometric normalization may amplify the noise. In the present study, cells with inappropriate boundary detection were excluded at the initial segmentation stage; therefore, sufficient binding analyses were achieved using both the *I*_T_ and *I*_T_/*I*_R_ values. These observations suggest that ratiometric analysis is not universally applicable but can be effective when the reference channel is stably acquired. Accordingly, the comparative use of *I*_T_ and *I*_T_/*I*_R_, while considering individual sample characteristics, is expected to lead to more reliable binding analysis.

### Web-based implementation and user-adjustable analysis settings

The DualCellQuant framework is designed as a parameterized system rather than a fixed pipeline, enabling users to modify analysis settings in accordance with specific imaging conditions and experimental objectives. The analysis method is available as a web-based interface, DualCellQuant. This platform accepts two types of microscopy images and assigns them as reference and target images, respectively. Beyond the ratiometric analysis between the two channels, our framework enables a flexible quantitative evaluation of signal changes relative to the reference images under various experimental conditions. The reference image can be flexibly selected, including membrane-localized fluorescence, bright-field images, or other fluorescence channels, depending on the analytical objectives. By offering these options through a web-based interface, the framework ensures reproducibility while remaining adaptable to a variety of experimental configurations. In this study, the parameter values and criteria represent a practical configuration optimized for the current application example, including cell inclusion/exclusion criteria, channel-wise masking strategy, and membrane-region definition. These settings are adjustable via the web-based interface, and users are encouraged to refine them when applying the tool to different cell types, signal-to-noise regimes, or microscopy setups. Notably, these parameter choices do not impose fundamental limitations on the framework itself but rather provide flexible options for robust membrane intensity quantification across diverse experimental contexts.

## Conclusion

In this study, we have developed an image analysis framework designed for the objective and reproducible quantification of fluorescence intensity within the plasma membrane region. By integrating AI-based automatic cell segmentation with EDT-based distance normalization, this framework facilitates consistent per-cell quantification without the need for user-defined region selection. Recent advancements in segmentation methods, such as Cellpose-SAM [8], enable the accurate extraction of cell boundaries without additional training, thereby providing a stable spatial reference for normalized intensity analysis across cells of varying shapes and sizes. The proposed method is implemented as a web-based interface, DualCellQuant, which supports flexible assignment of reference and target images and allows for quantitative evaluation under diverse experimental conditions. Due to its flexibility and robustness, the present framework is anticipated to be broadly applicable to biological imaging studies and bioanalytical applications beyond the analysis of cell surface fluorescence.

## Supplementary Information

Below is the link to the electronic supplementary material.

Supplementary Material 1

## Data Availability

The web application, DualCellQuant, and the resources generated in this study are available as follows: Web application: https://dna00.bio.kyutech.ac.jp/dualcellquant/. Source code: https://github.com/fuji3to4/DualCellQuant. Archived source code (Zenodo, v1.0.0): 10.5281/zenodo.18347379. Microscopy image dataset (Zenodo): 10.5281/zenodo.18321816.

## References

[1] E. DʼEste, G. Lukinavičius, R. Lincoln, F. Opazo, E.F. Fornasiero, Advancing cell biology with nanoscale fluorescence imaging: Essential practical considerations. Trends Cell. Biol. **34**, 671–684 (2024). 10.1016/j.tcb.2023.12.001 38184400 10.1016/j.tcb.2023.12.001

[2] C. Liu, X. Gao, J. Yuan, R. Zhang, Advances in the development of fluorescence probes for cell plasma membrane imaging. TrAC-Trends Anal. Chem. **133**, 116092 (2020). 10.1016/j.trac.2020.116092

[3] Z. Duan, K. Li, W. Duan, J. Zhang, J. Xing, Probing membrane protein interactions and signaling molecule homeostasis in plants by Förster resonance energy transfer analysis. J. Exp. Bot. **73**, 68–77 (2022). 10.1093/jxb/erab445 34610124 10.1093/jxb/erab445

[4] V. Kumar, P.K.C. Lakshman, T.K. Prasad, K. Manjunath, S. Bairy, A.S. Vasu, B. Ganavi, S. Jasti, N. Kamariah, Target-based drug discovery: Applications of fluorescence techniques in high throughput and fragment-based screening. Heliyon. **10**, e23864 (2024). 10.1016/j.heliyon.2023.e23864 38226204 10.1016/j.heliyon.2023.e23864 PMC10788520

[5] Y. Fu, X. Zhang, L. Wu, M. Wu, T.D. James, R. Zhang, Bioorthogonally activated probes for precise fluorescence imaging. Chem. Soc. Rev. **54**, 201–265 (2025). 10.1039/d3cs00883e 39555968 10.1039/d3cs00883e

[6] J. Adler, A. Huang, I. Parmryd, Find_plasma_membrane and measure_plasma_membrane: ImageJ macros for efficient identification of and measurements at and around the plasma membrane. SoftwareX. **24**, 101570 (2023). 10.1016/j.softx.2023.101570

[7] C. Stringer, T. Wang, M. Michaelos, M. Pachitariu, Cellpose: A generalist algorithm for cellular segmentation. Nat. Methods. **18**, 100–106 (2021). 10.1038/s41592-020-01018-x 33318659 10.1038/s41592-020-01018-x

[8] M. Pachitariu, M. Rariden, C. Stringer, Cellpose-SAM: Superhuman generalization for cellular segmentation. bioRxiv 2025.04.28.651001 (2025) 10.1101/2025.04.28.651001

[9] C.W. Wang, W.T. Lee, T.S. Su, A survey of deep learning methods on cell instance segmentation. Neural Comput. Appl. **37**, 11195–11264 (2025). 10.1007/s00521-025-11119-3

[10] O. Ronneberger, P. Fischer, T. Brox, *Medical image computing and computer-assisted intervention–—MICCAI 2015* (Springer, 2015), pp. 234–241

[11] A. Kirillov, E. Mintun, N. Ravi, H. Mao, C. Rolland, L. Gustafson, T. Xiao, S. Whitehead, A.C. Berg, W.Y. Lo, P. Dollár, R. Girshick, arXiv 2304.02643 (2023) 10.48550/arXiv.2304.02643

[12] P.E. Danielsson, Comput. Graph Image Process. Pu. **14**, 227–248 (1980). 10.1016/0146-664X(80)90054-4

[13] L.A. Banaszynski, C.W. Liu, T.J. Wandless, Characterization of the FKBP⊙ Rapamycin⊙ FRB ternary complex. J. Am. Chem. Soc. **127**, 4715–4721 (2005). 10.1021/ja043277y 15796538 10.1021/ja043277y

[14] D.M. Spencer, T.J. Wandless, S.L. Schreiber, G.R. Crabtree, Controlling signal transduction with synthetic ligands. Science. **262**, 1019–1024 (1993). 10.1126/science.7694365 7694365 10.1126/science.7694365

[15] J. van Unen, N.R. Reinhard, T. Yin, Y.I. Wu, M. Postma, T.W.J. Gadella, J. Goedhart, Plasma membrane restricted RhoGEF activity is sufficient for RhoA-mediated actin polymerization. Sci. Rep. **5**, 14693 (2015). 10.1038/srep14693 26435194 10.1038/srep14693 PMC4592971

[16] A.V. Karginov, F. Ding, P. Kota, N.V. Dokholyan, K.M. Hahn, Engineered allosteric activation of kinases in living cells. Nat. Biotechnol. **28**, 743–747 (2010). 10.1038/nbt.1639 20581846 10.1038/nbt.1639 PMC2902629

[17] K. Hirano, S. Sueda, A fluorescence-based binding assay for proteins using the cell surface as a sensing platform. Anal. Sci. **40**, 563–571 (2024). 10.1007/s44211-023-00476-5 38091253 10.1007/s44211-023-00476-5

[18] A. Meijster, J.B.T.M. Roerdink, W.H. Hesselink, *Mathematical morphology and its applications to image and signal processing* (Kluwer Academic, Boston, 2005), pp. 331–340

[19] A. Savitzky, M.J.E. Golay, Smoothing and differentiation of data by simplified least squares procedures. Anal. Chem. **36**, 1627–1639 (1964). 10.1021/ac60214a047

[20] J. Chen, X.F. Zheng, E.J. Brown, S.L. Schreiber, Identification of an 11-kDa FKBP12-rapamycin-binding domain within the 289-kDa FKBP12-rapamycin-associated protein and characterization of a critical serine residue. Proceedings of the National Academy of Sciences. USA 92, 4947–4951 (1995) 10.1073/pnas.92.11.4947 10.1073/pnas.92.11.4947 PMC41824 7539137

