# Supplementary material for: Dual-color image analysis for quantifying fluorescence intensity in plasma membrane region of cells
Source: Anal Sci. 2026 Apr 14;42(7):577–88. doi: 10.1007/s44211-026-00908-y (PMC13294165; doi:10.1007/s44211-026-00908-y)
Supplement: Supplementary file 1 — Supplementary Material 1 [file 44211_2026_908_MOESM1_ESM.pdf]

Supplementary Information

Dual-color image analysis for quantifying fluorescence intensity in plasma membrane region of cells

Satoshi Fujii*, Keita Takaki, and Shinji Sueda

Department of Bioscience and Bioinformatics, Kyushu Institute of Technology, 680-4 Kawazu, Iizuka 820-8502, Japan

*To whom correspondence should be addressed.

E-mail: sfujii@bio.kyutech.ac.jp

Contents

- Procedures for construction of the expression plasmid for FKBP12-TM-mApple
- Procedures for construction of the expression plasmid for Cys-FRB
- **Fig. S1**: Pixel-level representation of the Euclidean distance transform (EDT) used for the radial distance calculation.
- **Fig. S2**: Radial fluorescence intensity profiles of the cells at different Fluo-FRB concentrations.
- **Fig. S3**: Binding curves between FKBP12 and FRB calculated based on the fluorescence intensity of the entire cell region.
- **Table S1**: Dissociation constants (K_D) and fitting statistics obtained using fluorescence intensities from the entire cell region.

Procedures for construction of the expression plasmid for FKBP12-TM-mApple

The expression plasmid for FKBP12-TM-mApple in mammalian cells was constructed using pBCCP-TM-mApple prepared in the previous study [1] and pmCheery-FKBP12 obtained from Addgene (Addgene plasmid # 67900) [2]. The desired plasmid was constructed by replacing the gene of BCCP in pBCCP-TM-mApple with that of FKBP12 in pmCheery-FKBP12. For this purpose, the upstream and downstream restriction enzyme sites of the BCCP gene in pBCCP-TM-mApple were first replaced with alternative restriction enzyme sites. Specifically, the HindIII site located upstream of the BCCP gene was replaced with the MluI site by inverse PCR-based mutagenesis with following primers: 5’-ACGCGTGGTGAAATCCTGTCTCCCATGCAGG-3’ and 5’-TCCTCGATCCTTGTACAGCTTCCTTTGGAAC-3’ (the MluI site is underlined). Likewise, the BamHI site located downstream of BCCP gene was replaced with the XbaI site by inverse PCR-based mutagenesis with following primers: 5’-TCTAGAGGAGGCGCTGTGGGCCAGGAC-3’ and 5’- CTTGATGATGAGGAGCAGATCGCCTTTCTTAACG-3’ (the XbaI site is underlined). The resulting plasmid was named p(MluI)BCCP(XbaI)-TM-mApple. On the other hand, the FKBP12 gene was amplified by PCR using pmCheery-FKBP12 as a template and the following primers: 5’ ACGCGTATGGGAGTGCAGGTGGAAACC-3’ and 5’- TCTAGATTCCATTTTTAGAAGCTCCACATCG-3’ (MluI and XbaI sites are underlined). Following the attachment of an adenine nucleotide to the 3’ end of the PCR product, it was inserted into the pTAC-2 vector, and the FKBP12 gene was then cut out from the resulting vector by digestion with MluI/XbaI. The obtained fragment was inserted into p(MluI)BCCP(XbaI)-TM-mApple digested with MluI/XbaI to give a plasmid, pFKBP12-TM-mApple.

References

1. K. Hirano, S. Sueda, Anal. Sci. (2024) https://doi.org/10.1007/s44211-023-00476-5
2. J. van Unen, N.R. Reinhard, T. Yin, Y.I. Wu, M. Postma, T.W. Gadella, J. Goedhart. Sci. Rep. (2015) https://doi.org/10.1038/srep14693

Procedures for construction of the expression plasmid for Cys-FRB

The expression plasmid for Cys-FRB in in *Escherichia coli* was constructed by modifying the FRB gene derived from pEGFP-FRB (Addgene plasmid # 25919) [1]) and inserting it into the pET21a vector. Specifically, the FRB gene, in which a codon for a cysteine residue was inserted after the initiation codon, was amplified by PCR using pEGFP-FRB as a template and the following primers: 5'-ATACATATG**TGT**ATCCTCTGGCATGAGATGTGG-3' and 5'-ATGCTCGAGCTTTGAGATTCGTCGGAACACATG-3' (NdeI and XhoI sites are underlined and the codon for the cysteine residue is shown in bold). The obtained PCR product was digested with NdeI/XhoI, and the resulting fragment was inserted into the pET21a vector digested with NdeI/XhoI to give a plasmid, pCys-FRB.

Reference

1. A.V. Karginov, F. Ding, P. Kota, N.V. Dokholyan, K.M. Hahn, Nat. Biotechnol. (2010) https://doi.org/10.1038/nbt.1639

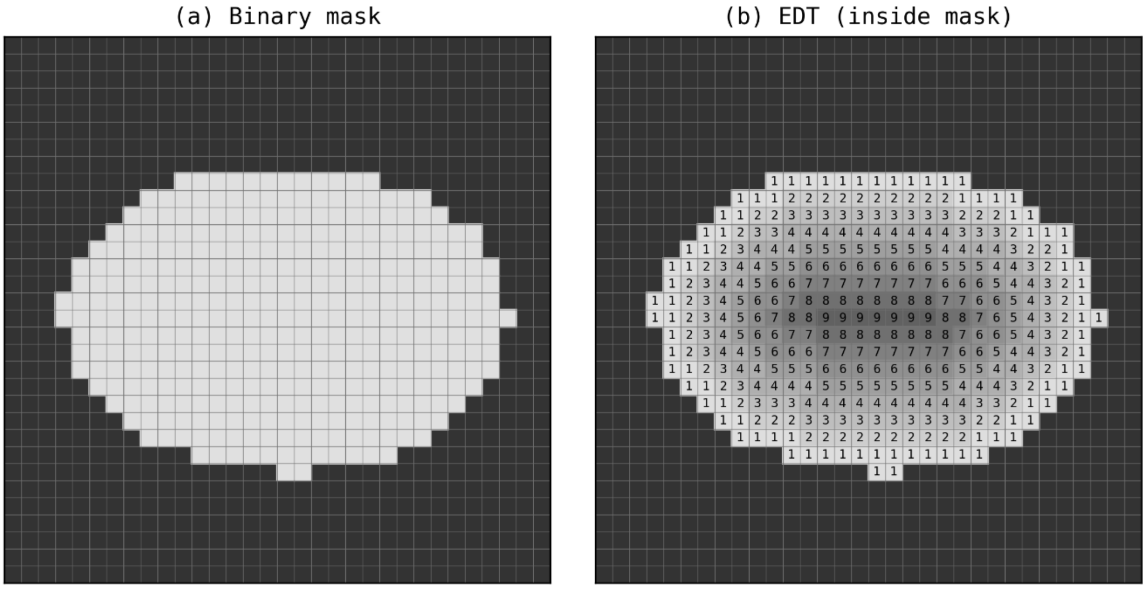

Fig. S1 Pixel-level representation of the Euclidean distance transform (EDT) used for the radial distance calculation. (a) Binary mask of the representative segmented cell. (b) Euclidean distance transformation was applied to the binary mask, where each pixel value represents the distance to the nearest point from the cell boundary. The pixel-wise EDT values are shown for clarity to illustrate how the distances from the cell boundary are quantified prior to normalization.

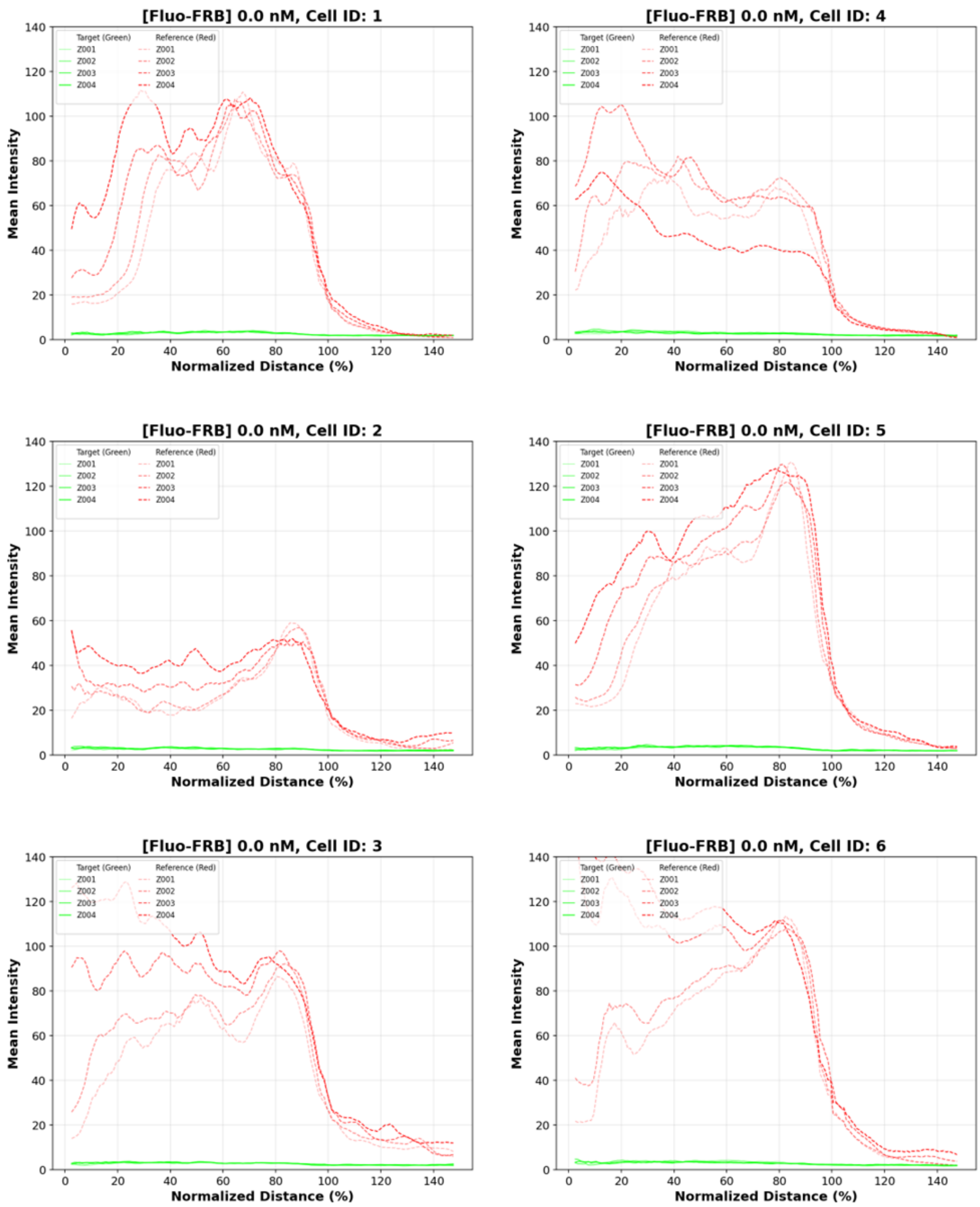

Fig. S2 Radial fluorescence intensity profiles of the cells at different Fluo-FRB concentrations. Radial profiles of mean fluorescence intensity are shown from cell center to extracellular region for Cell IDs 1–6. Each page shows profiles obtained at a different Fluo-FRB concentration (0, 0.5, 1, 2, 5, 10, and 20 nM). Green solid lines represent the target channel (fluorescein), and red dashed lines represent the reference channel (mApple). Profiles from four z-slice images (Z001‑Z004) are shown for each condition.

Fig. S2 (continued)

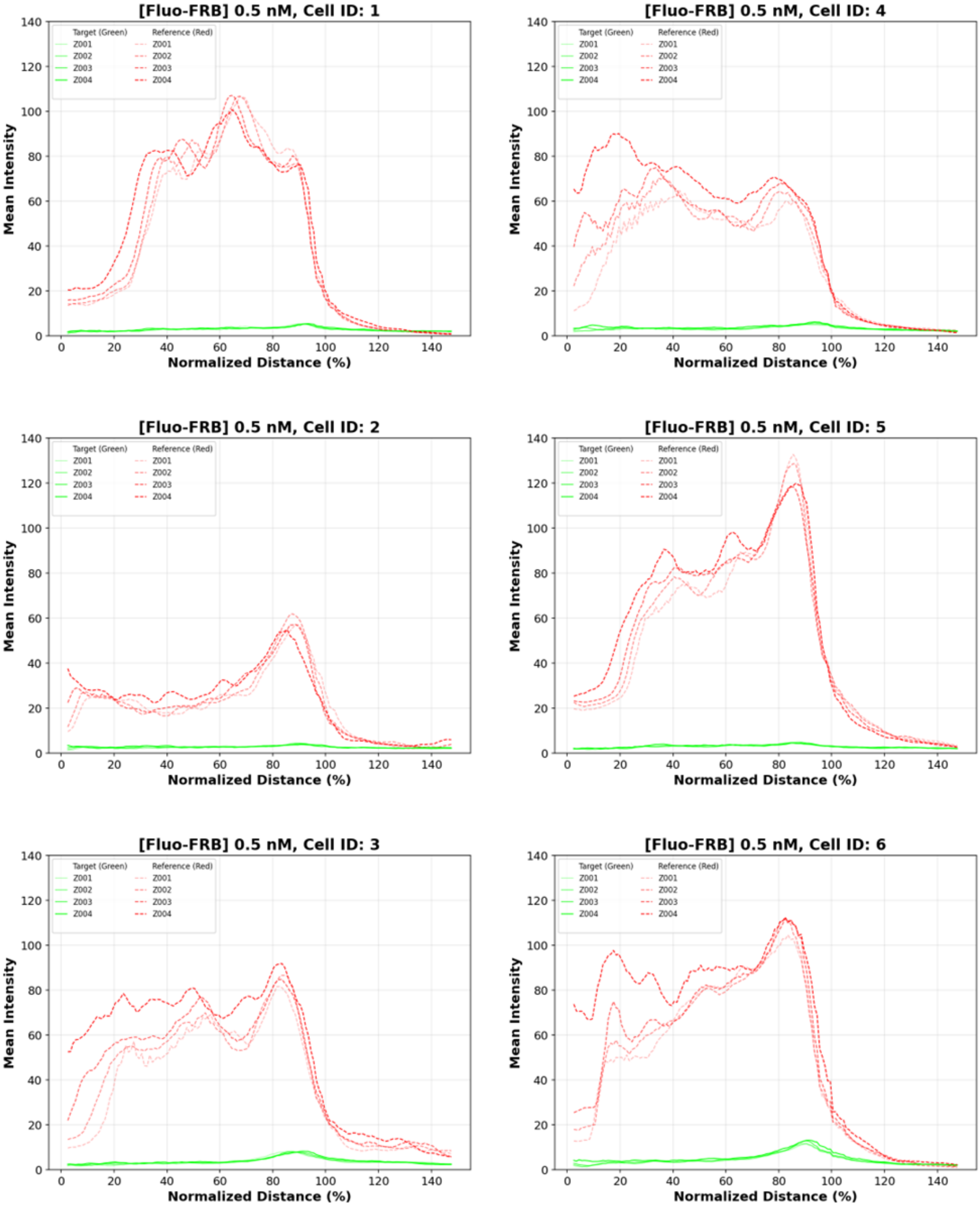

Fig. S2 (continued)

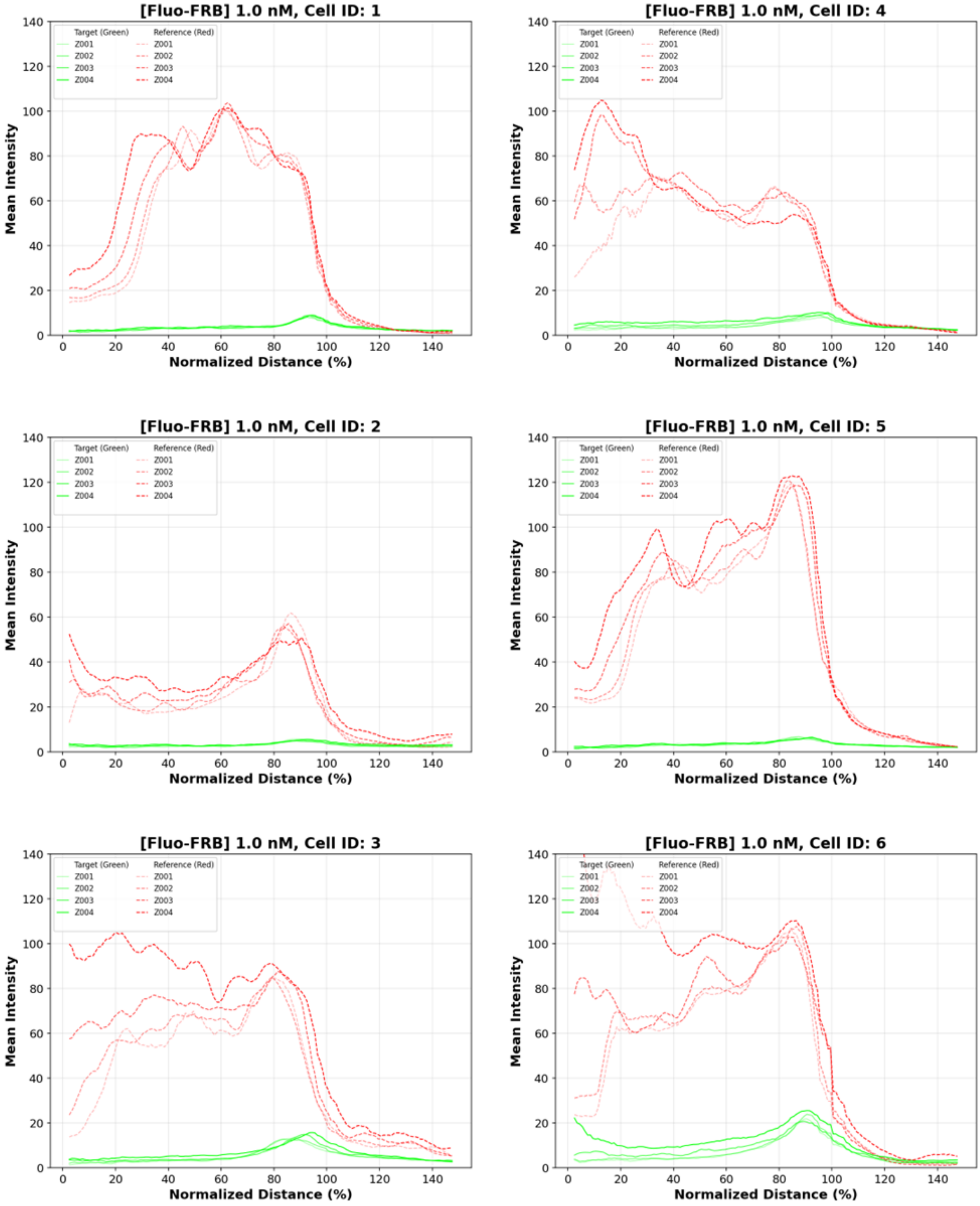

Fig. S2 (continued)

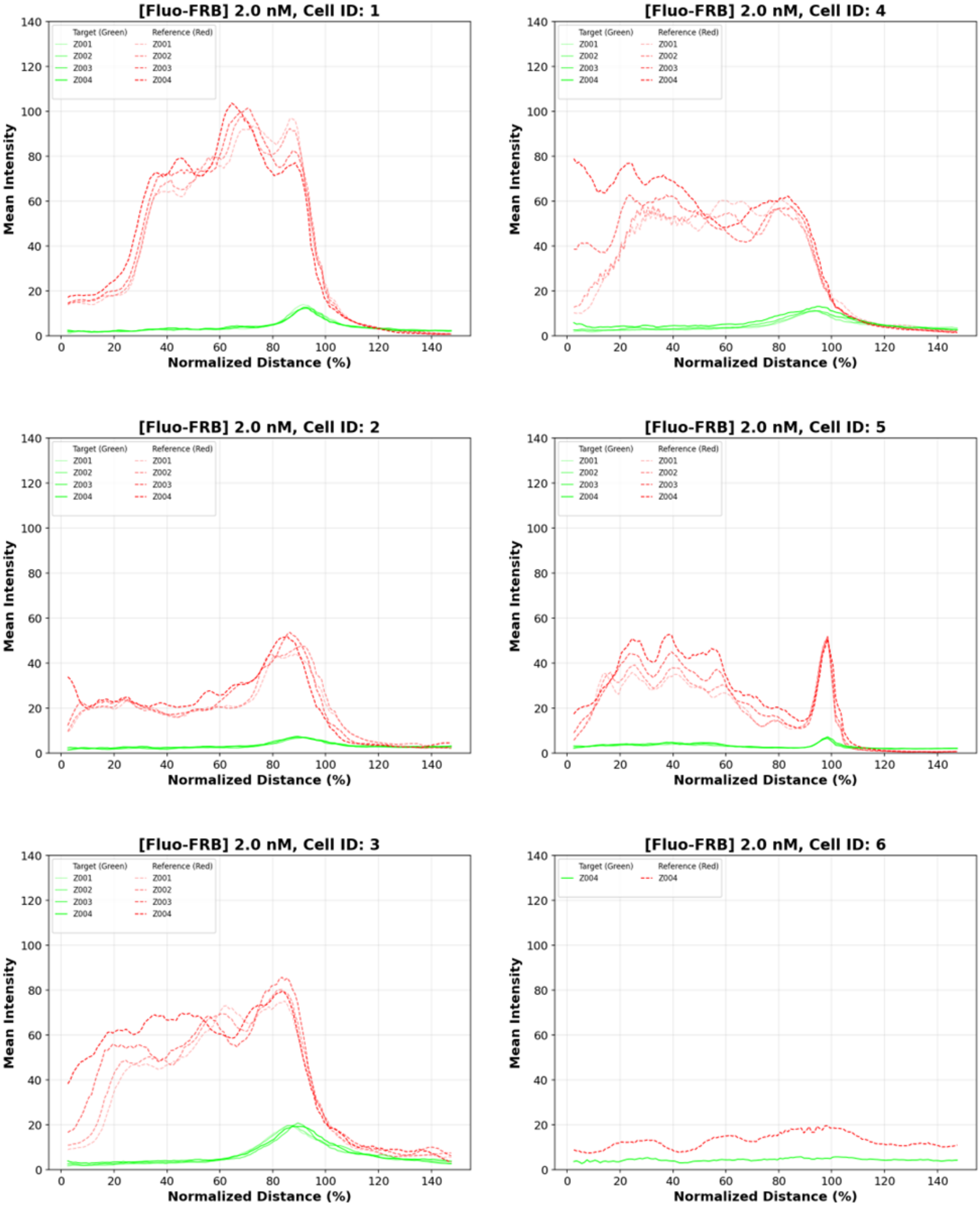

Fig. S2 (continued)

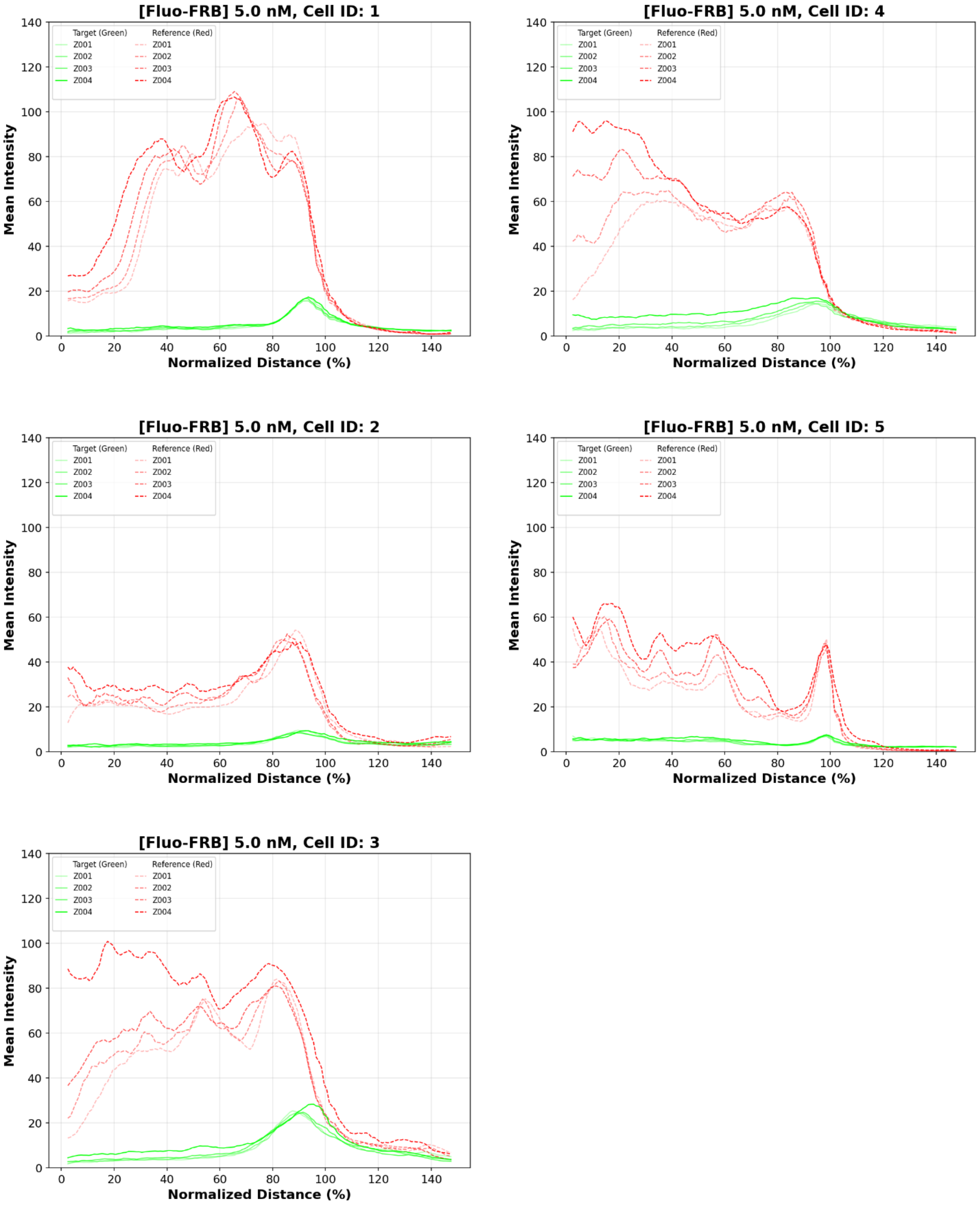

Fig. S2 (continued)

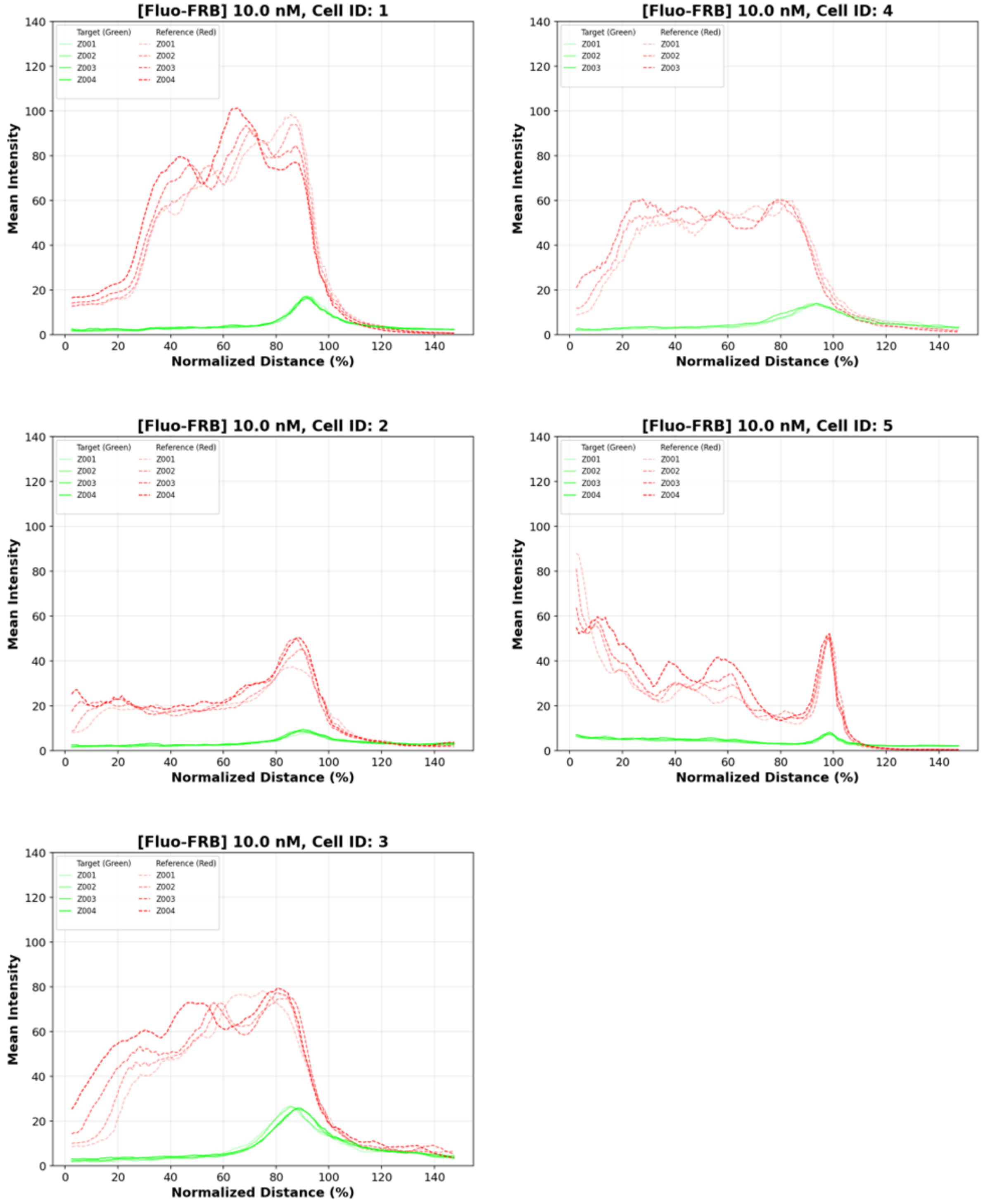

Fig. S2 (continued)

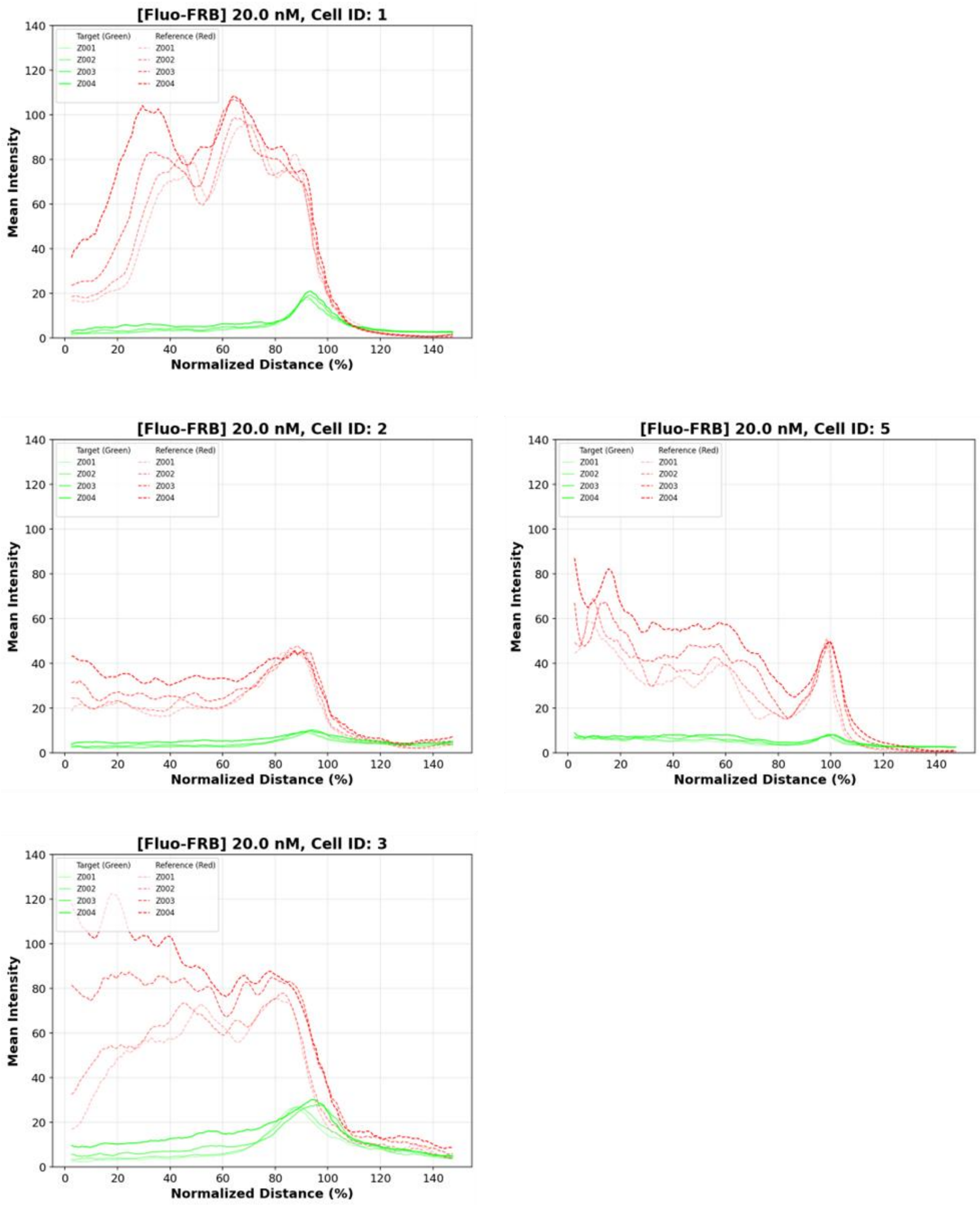

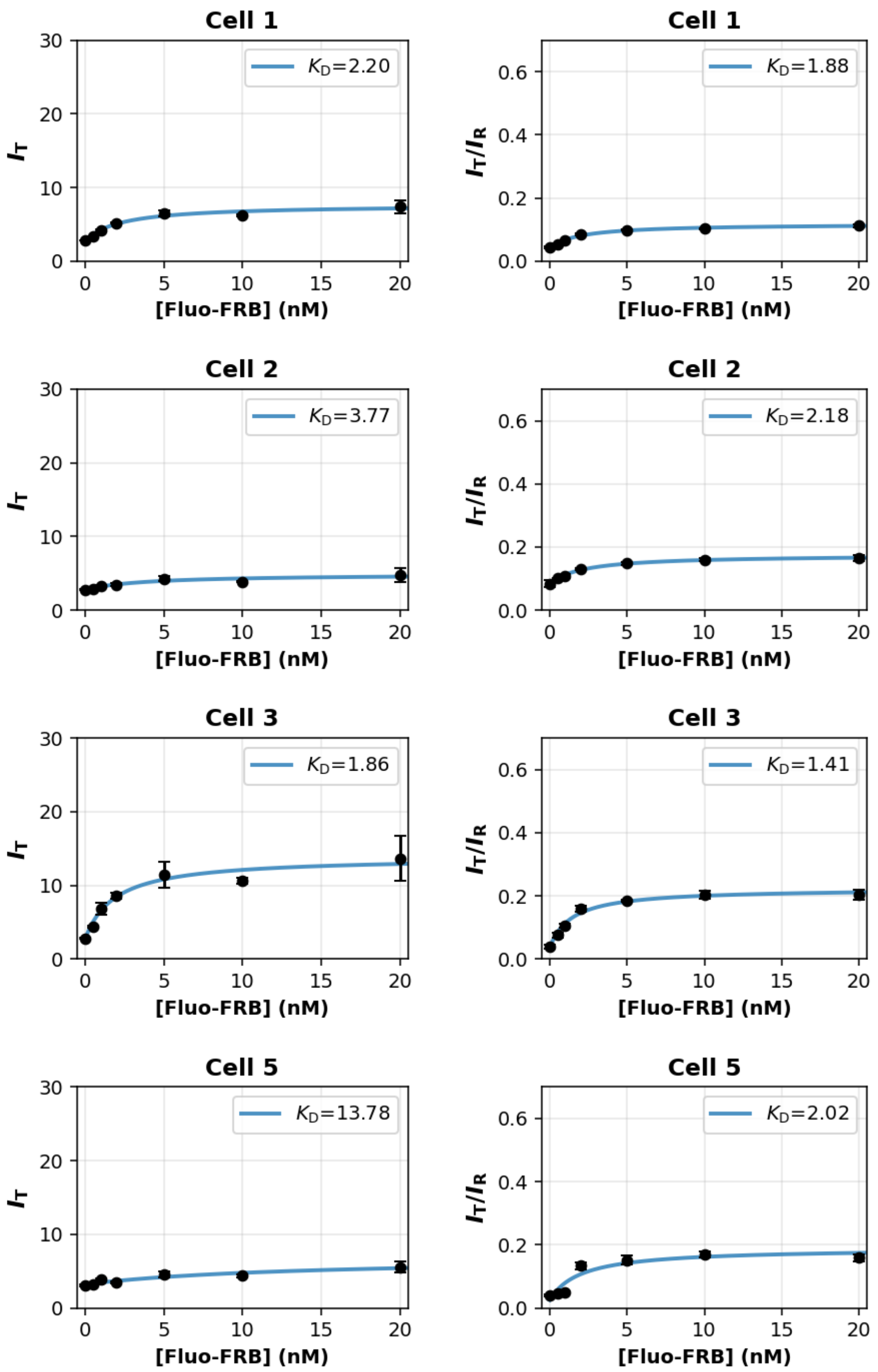

Fig. S3 Binding curves between FKBP12 and FRB calculated based on the fluorescence intensity of the entire cell region. Binding curves were generated on individual cells (Cell IDs 1, 2, 3, and 5) by plotting the mean fluorescence intensity from the target channel (I_T) or the ratio (I_T/I_R) of I_T to that from the reference channel (I_R) against the Fluo-FRB concentration; the former curves are shown in the left column and the latter curves are shown in the right column. Each curve was fitted to the Langmuir equation (2). Data points represent mean values obtained from four z-slice images for each Fluo-FRB concentration, and the error bars indicate standard deviations. Solid lines indicate nonlinear regression fits, and the estimated dissociation constants (K_D) in nanomolar concentration are shown in each panel.

Table S1 Dissociation constants (K_D) and fitting statistics obtained using fluorescence intensities from the entire cell region[a].

	I_T			I_T/I_R		
Cell ID	K_D (nM)[b]	SE[b] of K_D	R^{2b}	K_D (nM)[b]	SE[b] of K_D	R^{2b}
1	2.20	0.80	0.969	1.88	0.38	0.989
2	3.77	2.90	0.899	2.18	0.29	0.995
3	1.86	0.74	0.961	1.41	0.32	0.985
5	13.78	16.20	0.882	2.02	1.41	0.891
means ± SD[c]	5.40 ± 5.65			1.87 ± 0.33		

[a]Langmuir isotherm fitting was performed on a per-cell basis using either the mean fluorescence intensity from the target channel (I_T) or the ratio (I_T/I_R) of I_T to that from the reference channel (I_R) as the binding metric.

[b]K_D values, standard errors of K_D, and coefficients of determination (R^2) were estimated using nonlinear regression analysis of the binding curves shown in Fig. S3.

[c]The means ± standard deviations from data on four cells are shown.
